# Polypropylene Contamination in Post-Consumer Polyolefin Waste: Characterisation, Consequences and Compatibilisation

**DOI:** 10.3390/polym13162618

**Published:** 2021-08-06

**Authors:** Erdal Karaagac, Mitchell P. Jones, Thomas Koch, Vasiliki-Maria Archodoulaki

**Affiliations:** Institute of Materials Science and Technology, Faculty of Mechanical and Industrial Engineering, Technische Universität Wien, 1060 Vienna, Austria; erdal.karaagac@tuwien.ac.at (E.K.); mitchell.jones@tuwien.ac.at (M.P.J.); thomas.koch@tuwien.ac.at (T.K.)

**Keywords:** post-consumer waste, mechanical recycling, polyethylene, polypropylene, contamination, composition, tensile properties, impact properties, compatibilisation

## Abstract

Plastic recycling strikes a balance between functional, mass producible products and environmental sustainability and is pegged by governments for rapid expansion. However, ambitious targets on recycled material adoption across new markets are at odds with the often heterogenous properties of contaminated regranulates. This study investigated polypropylene (PP) contamination in post-consumer low-density polyethylene (PE-LD) and mixed polyolefin (PO) regranulates. Calibration curves were constructed and PP content, its effect on mechanical properties and property recovery in compatibilised material assessed. FT-IR band ratios provided more reliable estimations of PP content than DSC melt enthalpy, which suffered considerable error for PP copolymers. PE-LD regranulates contained up to 7 wt.% PP contamination and were considerably more brittle than virgin PE-LD. Most mixed PO regranulates contained 45–95 wt.% PP and grew more brittle with increasing PP content. Compatibilisation with 5 wt.% ethylene-based olefin block copolymer resulted in PE-LD blends resembling virgin PE-LD and considerable improvements in the properties of mixed PO blends. These results illustrate the prevalence of PP in recycled PE, challenges associated with its quantification, effect on mechanical properties, and compatibilisation viability, thereby representing an important step towards higher quality regranulates to meet the recycling demands of tomorrow.

## 1. Introduction

With growing emphasis and legislative action on improving environmental sustainability, plastic recycling has become a forerunner in the race to optimise waste management practices, reduce reliance on fossil fuels and adopt closed loop circular economy principles across the globe. The European Commission aims to increase the use of recycled plastics in new products to 10 million tons/year by 2025 [1] and recycle 55% of all plastic packaging waste across the EU by 2030 [2]. Polyolefins (PO), such as polyethylene (PE) and polypropylene (PP), are popular packaging materials and represent more than half of the 29 million tons of plastic waste collected in the EU each year [3,4]. Such numbers very effectively illustrate the importance PO will play in meeting new recycling targets. However, to do so, the use of these recycled plastics will need to be expanded to new applications for which recycled material hasn’t traditionally been suitable due to issues with material properties stemming from contamination [4].

Recycled plastics often contain both inorganic and polymer-based contamination, which results in mechanical properties that diverge from application-specific targets and limit their use to sectors utilising lower grade materials, such as agriculture and construction [5]. This contamination often results from flawed sorting practices, which struggle to economically separate materials with very similar characteristics, such as polymers of very similar density [6]. PP is a common contaminant in both low-density polyethylene (PE-LD) and mixed PO regranulates [7]. Several recent studies generically characterise selected PE regranulates, providing differential scanning calorimetry (DSC) thermograms and Fourier transform infrared (FT-IR) spectra, as well as thermal degradation and mechanical properties, such as tensile, impact, fracture toughness, and hardness [4,8,9,10]. The effect of contamination on recycled polyolefins, including that from other polyolefins, and methodologies for the identification of such contaminants are also thoroughly documented in the literature [11,12,13,14,15,16]. However, a structured investigation centered on the characterisation of the PP constituent of PP contaminated PE blends, its effect on mechanical properties, and improvements possible through compatibilisation is clearly lacking.

This study aimed to investigate PP contamination in post-consumer PE-LD and mixed PO regranulates using the PP content as a base parameter and reference point for analysis rather than arbitrarily characterising these very heterogenous and often incomparable materials. DSC-based melt enthalpy and FT-IR band ratios were used to construct calibration curves to estimate blend PP content across a range of different PP types and their reliability contrasted. Tensile and tensile impact mechanical properties were then assessed and the influence of the PP content on these properties investigated. Finally, the regranulates were compatibilised to improve their mechanical properties and achieve a viable recycled substitute for virgin PE.

## 2. Materials and Methods

### 2.1. Materials

Film grade 290E low density polyethylene (PE-LD) (Dow Chemical Company, Midland, MI, U.S.A), blow moulding grade Hostalen GF4750 high density polyethylene (PE-HD) (LyondellBasell, Rotterdam, The Netherlands) and injection moulding grade HF700SA polypropylene (PP) (Borealis, Vienna, Austria) were purchased as reference materials and used to construct melting enthalpy and FT-IR band ratio calibration curves for calculating PP content in PE-LD and mixed PO blends. HD601CF film grade, HC600TF thermoforming grade and HA104E extrusion grade homopolymer PP, BA202E extrusion grade block- and RD208CF random copolymer PP were purchased from Borealis (Vienna, Austria) to investigate the effect of various types of PP common in regranulates on melting enthalpy and FT-IR band ratio calibration curves. P01-1,2,3, P03-1,2,3, and P05-1,2,3 PE-LD regranulates were provided by Walter Kunststoffe Regranulat (Gunskirchen, Austria). Purpolen PE Grau (PPE), Purpolen PP Grau (PPP), Dipolen H (DPH), Dipolen PP Grau (DPP), Dipolen S Grau (DPS), and Dipolen SP Grau (DSP) mixed PO regranulates were provided by MTM Regranulat (Niedergebra, Germany). INFUSE ethylene-based olefin block copolymer with glass transition and melting temperatures of −65 °C and 118 °C, respectively, and a tensile elongation at break of 1000% was provided by Dow Chemical Company (Midland, MI, USA) for use as compatibiliser. All materials were used as received.

### 2.2. Prepatation of Virgin, Regranulate and Compatibilised Blends

Blends of virgin PE-LD and PP used to construct melting enthalpy and FT-IR band ratio calibration curves for PE-LD regranulates were prepared using a HAAKE MiniLab II twin-screw extruder (Thermo Scientific, Waltham, MA, USA) running at 180 °C with a screw speed of 100 rpm and dwell time of 5 min. These lower temperature conditions were selected to minimize material degradation during extrusion. PP contents of 2, 5, 8, 10, 12, 15, 20, and 25 wt.% were weighed and hand mixed with PE-LD prior to extrusion. Melt mass-flow rates were calculated in accordance with ISO 1133-1:2011 [17].

Virgin PE-HD and PP blends were used to construct melting enthalpy and FT-IR band ratio calibration curves for mixed PO regranulates. These were prepared using an Extron-Mecanor SWL0914-1 single screw extruder (Toijala, Finland) with a nozzle temperature of 200 °C running at 75 rpm. PE-HD blends with a PP content of 2, 8, 10, 20, 30, 40, 50 60, 70, 80, 90, 92, 95, and 98 wt.% were weighed, hand mixed and extruded twice. Pure PE-HD and PP references were also prepared under the same conditions. An overview of the calibration curves used in this study, the materials, and sample compositions used to generate them and the regranulates subsequently assessed with each curve, is provided in Table 1.

PE-LD (P01, P03 and P05) and mixed PO (PPE, PPP, DPH, DPP, DPS and DSP) regranulates were again prepared using a HAAKE MiniLab II twin-screw extruder (Thermo Scientific, Waltham, MA, USA). Extruded plastic was collected from the die, cut into small pieces and compression moulded into sheets at 190 °C using a Collin P 200 P laboratory press. Preheating was completed at 150 °C and 8 bar (hydraulic press pressure) for 10 min, heating from 150–190 °C at 22 bar for 8 min, followed by compression at 190 °C and 30 bar for 5 min. Samples were then cooled from 190 °C to 30 °C over 20 min at 10 K/min and 35 bar. Blends compatibilised with 5 wt.% ethylene-based olefin block copolymer were prepared in the same way. The compatibiliser and its content (5 wt.%) were selected based on an extensive study of PE-HD compatibilisation, recommending its use in quantities of 4–8 wt.% [7].

### 2.3. Melting Enthalpy and FT-IR Band Ratio Characterisation and Calibration Curve Generation

Melting enthalpy was assessed using a TA Instruments Q 2000 differential scanning calorimeter (DSC) (New Castle, DE, USA). An ~8 mg sample mass of each polymer blend was deposited in an alumina testing pan and sealed. Samples were heated to 200 °C at 10 K/min, cooled at the same rate to room temperature, and then reheated under the same heating conditions as previously described. A nitrogen atmosphere was maintained at all times using a flow rate of 50 mL/min. The melting enthalpy $\Delta H_{m}$ of the second heating run was analysed using TA Instruments Universal Analysis 2000 (v. 4.5A, b. 4.5.0.5). Analysis was based on four replicate specimens for each sample type.

The use of calibration curves based on DSC melting enthalpy to calculate the composition of polyolefin blends is documented in the literature [13,14,15,16]. Melting enthalpy-based calibration curves were constructed based on the known PP content of the series of virgin PE-LD/PP and PE-HD/PP blends (X axis) and the melting enthalpy (J/g) defined as the area under the melting peak on the second heating run (Y axis). A linear fit was applied to the resulting points using OriginPro 2019b (v. 9.6.5.169) and the equation noted. The PP content of PE-LD and mixed PO regranulates could then be calculated by measuring their melting enthalpies using DSC and substituting these values into the equation to provide a solution.

IR spectra were recorded using a Bruker TENSOR 27 Fourier transform infrared (FT-IR) instrument in attenuated total reflection (ATR) mode. Three spectra were recorded from different portions of four individual samples to verify homogeneity. Spectra were recorded from 4000 to 400 cm^−1^.

FT-IR band ratio-based calibration curves enabling the calculation of the PP content in PE-LD regranulates were constructed based on the absorbance bands (amplitude) of the series of virgin PE-LD/PP blends of known composition at 1376 cm^−1^ and 1461 cm^−1^. This process is documented in ASTM D7399-18:2018 [18] and the literature [13,15,19]. The known PP content of each virgin blend (X axis) was plotted against the ratio of these bands (1376 cm^−1^/1461 cm^−1^) (Y axis), a linear fit applied to the resulting points, the equation noted and used as previously described. The same procedure was completed for the series of virgin PE-HD/PP blends to produce a calibration curve for mixed PO regranulates with the only exception that the absorbance (amplitude) at the bands 720 cm^−1^ and 1168 cm^−1^ were used in the ratio 1168 cm^−1^/(1168 cm^−1^ + 720 cm^−1^).

### 2.4. Tensile (Impact) Mechanical Testing of the Virgin, Regranulate and Compatiblised Blends

Dog bone shaped tensile test specimens were cut from compression moulded sheets (1.8–1.9 mm thick) according to type 5A, ISO 572-2:2012 [20]. Seven replicate tests were performed for each sample type at 23 °C and a testing velocity of 10 mm/min using a Zwick 050 universal testing system equipped with a 1 kN load cell and extensometer. Tensile modulus and elongation at break were calculated using the ZwickRoell testXpert III software.

It is important to note that a constant speed of 10 mm/min was used over the entire tensile testing range rather than testing as two distinct segments as suggested in ISO 572-2:2012. The strain rate calculated based on the narrow parallel part of the specimen (0.4 min^−1^) is subsequently 40 times higher than the strain rate of the special ‘modulus segment’ described in ISO 572-2:2012 (0.01 min^−1^).

Tensile impact test specimens were cut from compression moulded sheets (1.1–1.2 mm thick) according to method A, ISO 8256:2004 [21]. Seven replicate tests were performed for each sample type at 23 °C using an Instron CEAST 9050 impact pendulum equipped with a 2 J hammer and 15 g crosshead mass. Tensile impact strength ($a_{tN}$, kJ/m^2^) was calculated based on the corrected impact work ($E_{c}$, J), distance between notches (x, mm), and thickness of the narrow parallel test specimen section (h, mm) (Equation (1)).

(1) $$a_{tN}=\frac{E_{c}}{x\cdot h}\cdot 10^{3}$$

### 2.5. Thermal Degradation Analysis of the Regranulate

The thermal degradation properties and inorganic filler content of PE-LD (P05) and mixed PO (PPE, PPP, DPH, DPP, DPS, and DSP) regranulates were assessed using TA Instruments thermogravimetric analysis (TGA) Q500. Regranulate samples of ~10 mg were placed in an alumina crucible and heated from 30 to 600 °C at a heating rate of 10 K/min in an air atmosphere.

### 2.6. Morphological and Elemental Analysis of the Regranulate and Compitablised Blends

Scanning electron microscopy (SEM) imaging and energy dispersive X-ray spectroscopy (EDS) elemental analysis were used to investigate the fracture surfaces of the tensile impact tested PE-LD (P05) and mixed PO (PPE, PPP, DPH, DPP, DPS, and DSP) regranulate specimens and composition of inorganic regranulate residues following TGA, respectively. A Philips XL30 scanning electron microscope was used for the tensile impact tested samples while a ZEISS EVO 10 scanning electron microscope fitted with a ZEISS SmartEDS system was used for the inorganic regranulate residues.

## 3. Results and Discussion

### 3.1. Characterisation of Polypropylene Contamination in Post-Consumer Waste

**Figure 1:**
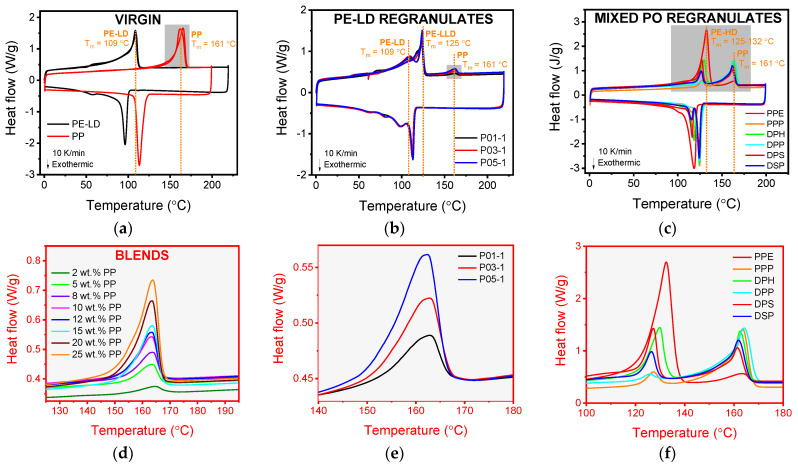
Differential scanning calorimetry (DSC) thermograms of (**a**) virgin PE-LD and polypropylene (PP) with (**d)** magnification of PP melting peak (T_m_ = 161 °C) illustrating melt enthalpy differences by blend PP content (2, 5, 8, 10, 12, 15, 20 and 25 wt.%), (**b**) P01,03,05 PE-LD regranulates with (**e**) magnification of PP melting peak (T_m_ = 161 °C) and (**c**) PPE, PPP, DPH, DPP, DPS and DSP mixed PO regranulates with (**f**) magnification of PE-HD (T_m_ = 125–132 °C) and PP (T_m_ = 161 °C) melting peaks.

DSC thermograms of virgin blends of known composition (neat PE-LD, PE-LD with 2, 5, 8, 10, 12, 15, 20, 25 wt.% PP and neat PP) indicated increasing area under the melting peak (melting enthalpy) at 161 °C, which is associated with PP, as the PP content increased (Figure 1). P01,03,05 PE-LD regranulates exhibited melting peaks at 109 °C and 125 °C, indicating that they primarily comprised PE-LD and PE-LLD [22]. An additional melting peak at 161 °C suggested a smaller quantity of PP present as contamination. The area under the PP melting peak varied considerably by PE-LD regranulate, ranging from the shortest and narrowest peak associated with P01 to the highest and widest peak for P05. Mixed PO regranulates (PPE, PPP, DHP, DPP, DPS and DSP) exhibited sizable melting peaks between 125–132 °C and at 161 °C attributable to PE-HD and PP, respectively. PPE exhibited the largest area under the melting peak associated with PE-HD and the smallest associated with PP, while PPP and DPP had the smallest area under the PE-HD melting peak and the largest under the PP peak.

**Figure 2:**
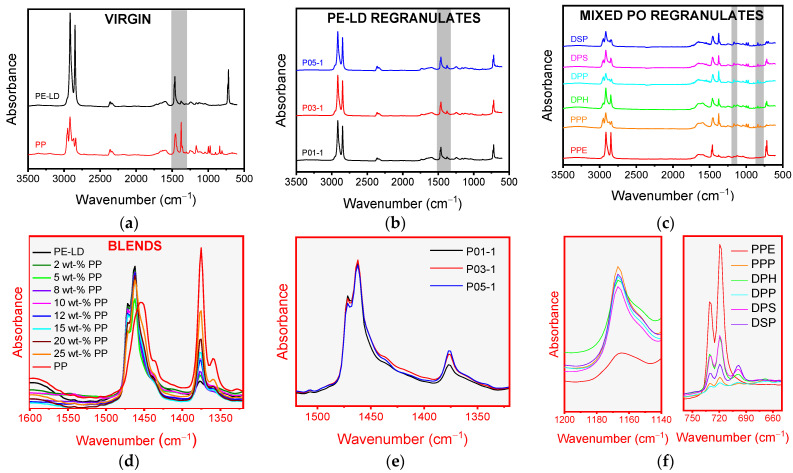
Fourier transform infrared (FT-IR) spectra of (**a**) virgin PE-LD and polypropylene (PP) with (**d**) magnification illustrating differences in the 1376 cm^−1^ (−CH_3_ plane bending) and 1461 cm^−1^ (−CH_2_ plane bending) bands by blend PP content (0, 2, 5, 8, 10, 12, 15, 20, 25 and 100 wt.%), (**b**) P01,03,05 PE-LD regranulates with (**e**) magnification of 1376 cm^−1^ (−CH_3_ plane bending) and 1461 cm^−1^ (−CH_2_ plane bending) bands and (**c**) PPE, PPP, DPH, DPP, DPS and DSP mixed PO regranulates with (**f**) magnification of 1168 cm^−1^ (−CH_3_ wagging) and 720 cm^−1^ (−CH_2_−rocking) bands.

FT-IR spectra of virgin blends of known composition indicated increasing band intensity at 1376 cm^−1^, which is associated with −CH_3_ plane bending, with increasing PP content (Figure 2). The band intensity at 1461 cm^−1^ associated with −CH_2_ plane bending simultaneously decreased. P01,03,05 PE-LD regranulates exhibited the same 1376 cm^−1^ band in addition to a light shoulder at 3200–3500 cm^−1^ associated with -OH and bands at 1565–1600 cm^−1^ resulting from–NH stretching. These bands could indicate traces of polyamide, polyester, or low molecular weight contaminants [23]. Mixed PO regranulates exhibited bands at 1168 cm^−1^ attributable to −CH_3_ wagging in PP and 720 cm^−1^ resulting from–CH_2_−rocking in PE-HD [24].

**Figure 3:**
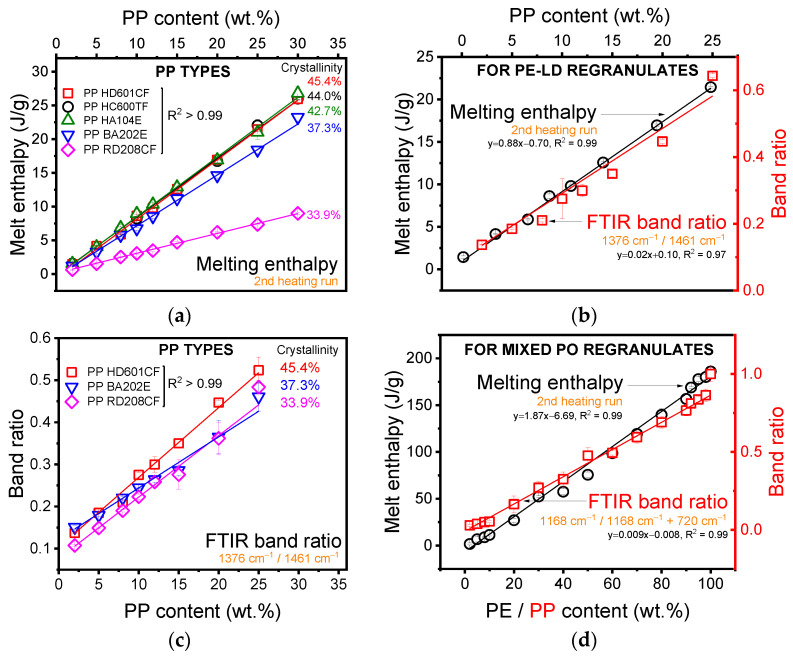
Calibration curves constructed based on (**a**) differential scanning calorimetry (DSC) melting enthalpy and (**c**) Fourier transform infrared (FT-IR) band ratios (1376 cm^−1^/1461 cm^−1^) of virgin PE-LD blends of known composition. DSC melting enthalpy-based curves vary considerably by polypropylene (PP) type (especially BA202E block- and RD208CF random copolymers) and crystallinity, while FT-IR band ratio-based curves are less affected. Calibration curves for determining PP content in (**b**) PE-LD and (**d**) mixed PO regranulates are constructed based on DSC melt enthalpy and FT-IR band ratios 1376 cm^−1^/1461 cm^−1^ for PE-LD regranulates and 1168 cm^−1^/(1168 cm^−1^ + 720 cm^−1^) for mixed PO regranulates.

Melting enthalpy-based calibration curves were almost identical for all virgin homopolymer PPs (HD601CF, HC600CF, and HA104E) used to simulate contaminants in PE-LD (Figure 3, Table 2). Block copolymer PP BA202E also exhibited a similar curve. However, random copolymer PP RD 208CF had a radically different gradient to the other curves. This discrepancy can be explained by the differing degree of crystallinity between the homopolymer (~43–45%), block–(~37), and random copolymer PPs (~34%), which affects the melting enthalpy. This makes quantification of the PP content in P01,03,05 PE-LD regranulates, potentially contaminated with any type of PP, challenging using the melting enthalpy. Estimated PP content in P01 PE-LD regranulate ranged from 3.4 to 8.9 wt.% using this method based on the different PP types.

FT-IR-based values were generally slightly higher than those based on the melting enthalpy, a phenomenon also noted in other studies [15] and most likely due to the migration of PP to the material surface during compression moulding. Homopolymer (HD601CF), block–(PP BA202E), and random copolymer (PP RD 208CF) PP calibration curves were much better aligned when using the FT-IR band ratio 1376 cm^−1^/1461 cm^−1^. The estimated PP content in PE-LD regranulate ranged from 3.7 to 5.3 wt.% across the different PP types. This makes FT-IR more suitable for estimating the PP content in P01,03,05 PE-LD regranulates. The similarity between the FT-IR band ratio-based calibration curves also allows the provision of a generic equation for calculating blend PP content independent of PP type (Equation (2)), which is useful since the type of PP contamination in regranulates is often unknown. This method is also advantageous as it is faster than DSC-based melting enthalpy experiments and is non-destructive.

(2) $$y=0.014x+0.098,\;R^{2}=0.91$$

Generic PP calibration curves for P01,03,05 PE-LD regranulates based on both melting enthalpy and FT-IR band ratios are provided in Figure 3. Similar curves are provided for mixed PO regranulates based on the melting enthalpy and FT-IR bands 720 cm^−1^ and 1168 cm^−1^, expressed as 1168 cm^−1^/(1168 cm^−1^ + 720 cm^−1^), of a series of virgin PE-HD (melting enthalpy) and PP (FT-IR band ratio) blends of known composition. These calibration curves were used to calculate the PP content in P01,03,05 PE-LD regranulates and PPE, PPP, DPH, DPP, DPS, and DSP mixed PO regranulates (Table 3).

Discrepancies between PP content calculated based on melting enthalpy and FT-IR band ratios of PE-LD regranulates were <2 wt.%. PP content varied by up to ~2 wt.% between batches of the same PE-LD regranulate. P01 PE-LD regranulate contained 2.6–3.0% and 2.7–4.6 wt.% PP based on melting enthalpy and FT-IR band ratios, respectively. P03 regranulate contained more PP (5.6–5.9 wt.% and 6.5–7.2 wt.% based on melting enthalpy and FT-IR band ratios, respectively) than P01 but P05 regranulate clearly had the highest PP content with a calculated value of 4.6–6.1 wt.% based on melting enthalpy and 6.9–7.4 wt.% based on the more accurate FT-IR band ratios. P05 PE-LD regranulate was subsequently selected for further mechanical tests.

Discrepancies between calculated PP content based on melting enthalpy and FT-IR band ratios were much higher for mixed PO regranulates than PE-LD regranulates, ranging up to ~24 wt.% for DPH (70.1 wt.% calculated based on melting enthalpy compared to 46.1 wt.% based on FT-IR band ratios). These inconsistencies are attributable to DSC melting curve overlap resulting from the presence of PP block or random copolymer contaminants, which contain an ethylene fraction represented as a low temperature shoulder overlapping the PE peak [16]. PP content calculations based on FT-IR band ratios were accepted as more accurate than values based on melting enthalpy and are subsequently reported here. DPP and PPP mixed PO regranulates comprised almost entirely PP (94.9 wt.% and 87.5 wt.%, respectively), while DSP comprised 60 wt.% PP and DPH and DPS were a little less than half PP (46.1 wt.% and 44.5 wt.%, respectively). PPE contained just 5.5 wt.% PP.

### 3.2. Thermal Degradation Properties and Inorganic Content of Post-Consumer Waste

P05 PE-LD and PPE, PPP, DPH, DPP, DPS, and DSP mixed PO regranulates exhibited thermal degradation properties typical of PP and PE, indicating negligible aging or well-stabilized material (Supplementary Figure S1). Onset of thermal degradation was determined by the PP phase and further degradation behavior by the dominant blend component. PP rich regranulates were hence readily distinguishable from those which contained large quantities of PE. The inorganic residues of mixed PO regranulates were 0.9 wt.% for PPE, 1.8 wt.% for DPS, 2.1 wt.% for DSP, 2.2 wt.% for DPP, 2.3 wt.% for PPP, and 2.4 wt.% for DPH. P05 PE-LD regranulate had an inorganic residue of 2.8 wt.%. Inorganic residues mainly consisted of Si, Ca, Ti, Mg, Fe, S, Na, and Cl.

### 3.3. Effect of Polypropylene Contamination on Tensile and Tensile Impact Properties

**Figure 4:**
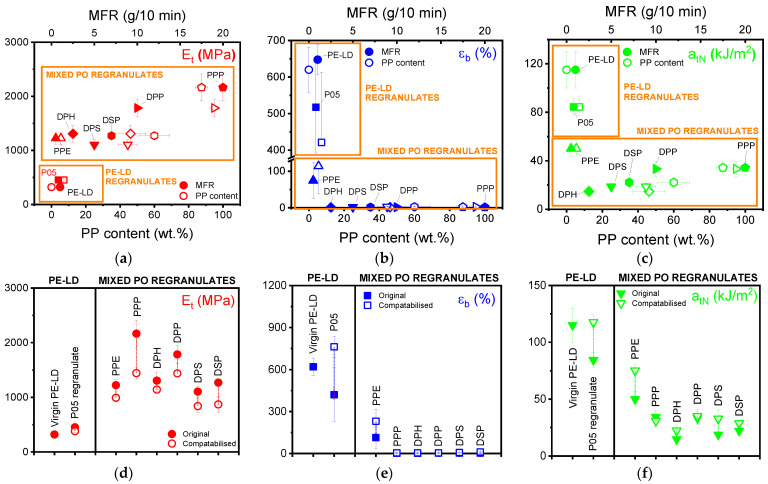
Effect of polypropylene (PP) content (hollow markers) and melt mass-flow rate (MFR, solid markers) on the (**a**) tensile modulus ($E_{t}$), (**b**) elongation at break ($\varepsilon_{f}$ ) and (**c**) tensile impact strength ($a_{tN}$ ) of PE-LD and mixed PO regranulates. Effect of 5 wt.% ethylene-based olefin block copolymer on the (**d**) $E_{t}$, (**e**) $\varepsilon_{f}$ and (**f**) $a_{tN}$ of PE-LD and mixed PO regranulates (solid markers prior to compatibilisation and hollow markers after).

P05 PE-LD regranulate had a higher tensile modulus ($E_{t}$) than virgin PE-LD (453 MPa compared to 320 MPa), a considerably lower elongation at break ($\varepsilon_{b}$) (421% compared to 620%) and tensile impact strength ($a_{tN}$) (84 kJ/m^2^ compared to 115 kJ/m^2^). These undesirable properties result from the ~7 wt.% PP contamination present in this PE-LD blend, which has a much higher $E_{t}$ than PE-LD and hinders miscibility and adhesion between blend components (Figure 4).

**Figure 5:**
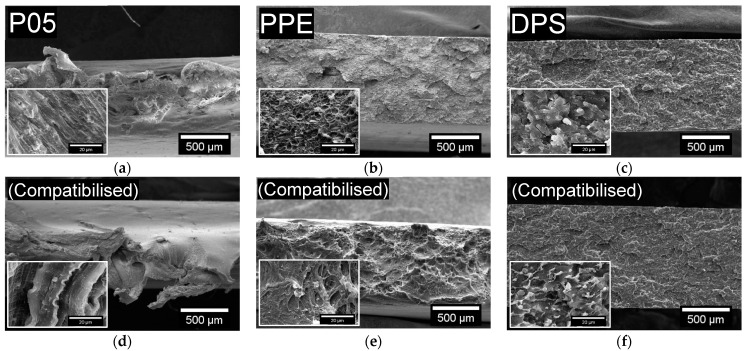
SEM micrographs of the tensile impact fracture surfaces of (**a**,**d**) P05 PE-LD, (**b**,**e**) PPE and (**c**,**f**) DPS mixed PO regranulates both before (**a**–**c**) and after (**d**–**f**) compatibilisation with 5 wt.% ethylene-based olefin block copolymer. P05 PE-LD regranulates demonstrated necking, especially in the compatibilised material, with shear yielding the primary deformation mechanism. PPE exhibited a brittle fracture surface with signs of energy dissipating effects, debonding and fibrillation before and extensive plastic deformation and strong fibrillation after compatibilisation. DPS fracture surfaces were macroscopically brittle both before and after compatibilisation, although compatibilised material did exhibit some regions of microplasticity. Higher magnification micrographs illustrating the phase morphology are provided in the insets.

Compatibilisation with 5 wt.% ethylene-based olefin block copolymer enhanced interfacial adhesion and provided an 81% improvement in the $\varepsilon_{b}$ of P05 (421% to 762%), a value 23% higher than even virgin PE-LD (762% compared with 620%). The compatibiliser also improved stress transfer between the phases resulting in a 39% increase in the $a_{tN}$ of P05 (84 kJ/m^2^ to 118 kJ/m^2^) and a comparable value to that of virgin PE-LD (115 kJ/m^2^). SEM micrographs indicated that the already present necking in P05 was increased post-compatibilisation with shear yielding the primary deformation mechanism in both samples (Figure 5). The $E_{t}$ of P05 simultaneously decreased with compatibilisation to 381 MPa, a 16% reduction which resulted in an $E_{t}$ just 19% higher than virgin PE-LD. This is due to the low $E_{t}$ of ethylene-based olefin block copolymer, which encapsulates the dispersed phase in the matrix and reduces the $E_{t}$ of the blends. Significantly, these results indicated that compatibilised P05 PE-LD regranulate exhibits competitive or better tensile properties than even virgin PE-LD films and can be considered a viable recycled substitute.

All mixed PO regranulates other than PPE exhibited high (≥~45 wt.%) PP contents and high melt mass-flow rates (MFR) (2.5−20.0 g/10 min) better suited to injection than compression moulding, which resulted in brittle tensile properties. Literature does, however, indicate similar properties in injection moulded samples suggesting a more general, moulding method independent sensitivity of $\varepsilon_{b}$ to the presence of mixtures of polymers with different molar mass distributions in recycled materials [25]. Blend $E_{t}$ increased with MFR and PP content, while $\varepsilon_{b}$ decreased violently at elevated MFRs and PP contents, indicating the sensitivity of this parameter to these factors. $a_{tN}$ was also sensitive to increasing MFR and PP content, dropping very quickly as PP content increased, but then recovering slightly at MFR ≥ 5 g/10 min and PP contents ≥ 60 wt.%.

PPP was the stiffest mixed PO regranulate with an $E_{t}$ of 2160 MPa, almost double that of DPS (1100 MPa), which had the lowest $E_{t}$. PPP’s stiffness resulted from its high PP content (~88 wt.%), which was approximately double that of DPS (~45 wt.%). Notably, DPP while also exhibiting a high $E_{t}$ (1780 MPa) wasn’t as stiff as PPP despite containing more PP (~95 wt.% compared to ~88 wt.%). PPE, DPH, DPS, and DSP all fell in the range of 1220–1310 MPa, despite PPE having a much lower PP content than the other mixed PO regranulates (~6 wt.% compared to ~45–60 wt.%). This low PP content did, however, endow PPE with an $\varepsilon_{b}$ 38–75 times higher than all other mixed PO regranulates (114% compared with 1.5–3.0%, respectively) and 1.5–3.5 times higher $a_{tN}$ (50.0 kJ/m^2^ compared to 14.7–34.4 kJ/m^2^).

The relatively high $E_{t}$ of mixed PO regranulates could potentially have been attributed to mineral fillers, such as calcium carbonate or talc. However, the low inorganic residues in the PE-LD and mixed PO regranulates (0.9–2.8 wt%) could be neglected due to the relatively high strain rate in the region of modulus determination and the high crystallinity of the samples resulting from the lower cooling rate used during compression moulding (10 K/min), as opposed to injection moulding.

Compatibilisation with 5 wt.% ethylene-based olefin block copolymer doubled the $\varepsilon_{b}$ of PPE (114% to 231%) and increased its $a_{tN}$ by 50% (50.1 kJ/m^2^ to 75.2 kJ/m^2^). SEM micrographs revealed that the previously brittle fracture surface of PPE, which showed signs of energy dissipating effects, debonding and fibrillation was, extensively plastically deformed following compatibilisation with strong fibrillation visible (Figure 5). Compatibilised PPP, DPH, DPP, DPS, and DSP all exhibited slightly higher $\varepsilon_{b}$ (~2–8%). DPS also experienced a considerable increase in $a_{tN}$ (14.1 kJ/m^2^) following compatibilisation but DPH, DPP and DSP were restricted to small improvements (1.8–7.6 kJ/m^2^), while the $a_{tN}$ of PPP decreased (34.3 kJ/m^2^ to 30.9 kJ/m^2^). Whether compatibilised or not, DPS fracture surfaces macroscopically appeared brittle, with improvements in the compatibilised material limited to some regions of microplasticity (Figure 5). Compatibilisation also resulted in a ~13–33% reduction in $E_{t}$ for all mixed PO regranulates. SEM micrographs of the tensile impact fracture surfaces of the other mixed PO regranulates are provided in Supplementary Figure S2.

## 4. Conclusions

PE-LD and mixed PO regranulates can be contaminated with any number of different PP types, which complicates blend characterisation and results in mechanical properties that diverge from application-specific targets. Variations in the crystallinity and ethylene fractions of PP copolymers affect the PP melting peak and cause low temperature shoulders that overlap the PE melting peak in DSC thermograms. This study found calibration curves constructed based on reference blends with known concentrations of virgin material and FT-IR band ratios more reliable in estimating the PP content of regranulates than those based on DSC melting enthalpy. P05 regranulate was the most highly contaminated PE-LD blend containing ~7 wt.% PP, which made it considerably stiffer and more brittle than virgin PE-LD. Most mixed PO regranulates contained 45–95 wt.% PP which also resulted in stiff and brittle tensile and tensile impact properties that only worsened with increasing PP content and MFR. Compatibilisation with as little as 5 wt.% ethylene-based olefin block copolymer considerably reduced the tensile modulus of all regranulates in addition to increasing their elongation at break and tensile impact strength. Compatibilised P05 PE-LD regranulates in fact exhibited comparable tensile and tensile impact properties to virgin PE-LD and could be a viable recycled substitute. These results demonstrate the prevalence of PP in PE regranulates, the challenges associated with its characterisation, and the significant detrimental effects that it has on tensile and tensile impact properties. Identification of this contamination and its treatment with low quantities of suitable compatibilisers could radically improve the quality of recycled plastic reentering the market across the globe, promoting consumer confidence and interest in recycled products and improved environmental sustainability.

## Figures and Tables

**Table 1:** Overview of the calibration curves utilised in this study, the materials and sample compositions used to generate them and the regranulates that were subsequently analysed using each respective calibration curve.

Calibration Curve	Material	Sample Compositions	Regranulates Analysed with Curve
PP type	PE-LD 290E	PE-LD with 2, 5, 8, 10, 12, 15, 20 and 25 wt.% PP	P01 (Table 2)
	PP 601CF		
	PP HC600TF		
	PP HA104E		
	PP BA202E		
	PP RD208CF		
PE-LD regranulate	PE-LD 290E	PE-LD with 2, 5, 8, 10, 12, 15, 20 and 25 wt.% PP	P01, P03, P05 (Table 3)
	PP HF700SA		
Mixed PO regranulate	PE-HD GF4750	PE-HD with 2, 8, 10, 20, 30, 40, 50 60, 70, 80, 90, 92, 95 and 98 wt.% PP	PPE, PPP, DPH, DPP, DPS, DSP (Table 3)
	PP HF700SA		

**Table 2:** Melt mass-flow rate (MFR, g/10 min) for 2.16 kg at 230 °C, degree of crystallinity (%) and calibration curve equations for virgin homopolymer, block- and random copolymer polypropylenes (PP) and calculated PP content for P01 PE-LD regranulate by PP type based on the melting enthalpy and FT-IR band ratio methods.

Method	Type	Material	MFR (g/10 min)	Crystallinity (%)	Equation	PP in P01 (wt.%)
Melting enthalpy	Homo	PP HD601CF	8.0	45.4 ± 1.5	y=0.88x−0.72	3.88 ± 0.26
		PP HC600TF	2.8	44.0 ± 1.6	y=0.87x−0.25	3.40 ± 0.09
		PP HA104E	0.8	42.7 ± 0.5	y=0.88x−0.35	3.47 ± 0.09
	Block	PP BA202E	0.3	37.3 ± 0.2	y=0.75x−0.41	4.15 ± 0.14
	Random	PP RD208CF	8.0	33.9 ± 0.1	y=0.29x+0.12	8.91 ± 0.39
FT-IR band ratio	Homo	PP HD601CF	8.0	45.4 ± 1.5	y=0.017x+0.10	3.69 ± 0.90
	Block	PP BA202E	0.3	37.3 ± 0.2	y=0.013x+0.11	3.85 ± 0.87
	Random	PP RD208CF	8.0	33.9 ± 0.1	y=0.015x+0.08	5.30 ± 0.90

**Table 3:** Melt mass-flow rate (MFR, g/10 min) for 2.16 kg at ^a^ 190 °C and ^b^ 230 °C, and calculated polypropylene (PP) content of PE-LD and mixed PO regranulates based on the melting enthalpy and FT-IR band ratio calibration curves.

Type	Material	MFR (g/10 min)	Calculated PP Content (wt.%)	
			Melting Enthalpy	FT-IR Band Ratio
PE-LD regranulate	Regranulate P01-1	0.8 ^a^	3.0 ± 0.9	2.7 ± 1.7
	Regranulate P01-2		3.1 ± 0.8	4.6 ± 0.2
	Regranulate P01–3		2.6 ± 0.8	4.5 ± 0.6
	Regranulate P03-1		5.9 ± 0.5	6.5 ± 0.1
	Regranulate P03-2		5.6 ± 0.5	7.2 ± 0.7
	Regranulate P03-3		5.7 ± 0.7	6.8 ± 0.3
	Regranulate P05-1		6.1 ± 2.8	7.3 ± 1.3
	Regranulate P05-2		6.4 ± 1.7	7.4 ± 0.2
	Regranulate P05-3		4.6 ± 1.3	6.9 ± 2.4
Mixed PO regranulate	Purpolen PE (PPE)	0.5 ^a^	6.9 ± 5.3	5.5 ± 1.2
	Purpolen PP (PPP)	20 ^b^	91.4 ± 0.8	87.5 ± 3.7
	Dipolen H (DPH)	2.5 ^a^	70.1 ± 1.9	46.1 ± 9.0
	Dipolen PP (DPP)	10 ^b^	93.8 ± 0.3	94.9 ± 2.3
	Dipolen S (DPS)	5 ^b^	67.9 ± 0.4	44.5 ± 5.5
	Dipolen SP (DSP)	7 ^b^	82.3 ± 0.6	60.0 ± 9.0

## Data Availability

The data presented in this study are available on request from the corresponding author.

## Supplementary Materials

The following are available online at https://www.mdpi.com/article/10.3390/polym13162618/s1. Figure S1: thermogravimetric analysis of P05 PE-LD and PPE, PPP, DPH, DPP, DPS and DSP mixed PO regranulates. Figure S2: SEM micrographs of the tensile impact fracture surfaces of PPP, DPH, DPP and DSP mixed PO regranulates.
