# Supplementary material for: Polypropylene Contamination in Post-Consumer Polyolefin Waste: Characterisation, Consequences and Compatibilisation
Source: Polymers (Basel). 2021 Aug 6;13(16):2618. doi: 10.3390/polym13162618 (PMC8401875; doi:10.3390/polym13162618)
Supplement: Supplementary file 1 [file polymers-13-02618-s001.zip › polymers-1289332-supplementary.pdf]

Electronic supplementary material for:

Polypropylene contamination in post-consumer polyolefin waste: characterisation, consequences and compatibilisation

Erdal Karaagac,[1] Mitchell P. Jones,[1] Thomas Koch[1] and Vasiliki-Maria Archodoulaki[1,*]

[1] Institute of Materials Science and Technology, Faculty of Mechanical and Industrial Engineering, TU Wien, Gumpendorferstrasse 7, Objekt 8, 1060 Vienna, Austria
* Corresponding authors. *Email address*: vasiliki-maria.archodoulaki@tuwien.ac.at

List of Figures

Figure S1. Thermogravimetric analysis of P05 PE-LD and PPE, PPP, DPH, DPP, DPS and DSP mixed PO regranulates. Thermal degradation properties were typical of PP and PE, indicating negligible aging or well-stabilized material. Onset of thermal degradation was determined by the PP phase and further degradation behaviour by the dominant blend component. PP rich regranulates (PPP, DPH, DPP, DPS and DSP) were hence readily distinguishable from those which contained large quantities of PE (P05 and PPE). The inorganic residues of mixed PO regranulates were 0.9 wt.% for PPE, 1.8 wt.% for DPS, 2.1 wt.% for DSP, 2.2 wt.% for DPP, 2.3 wt.% for PPP and 2.4 wt.% for DPH. P05 PE-LD regranulate had an inorganic residue of 2.8 wt.%. Inorganic residues mainly consisted of Si, Ca, Ti, Mg, Fe, S, Na and Cl.

Figure S2. SEM micrographs of the tensile impact fracture surfaces of PPP, DPH, DPP and DSP mixed PO regranulates. Higher magnification micrographs illustrating the phase morphology are provided in the insets.

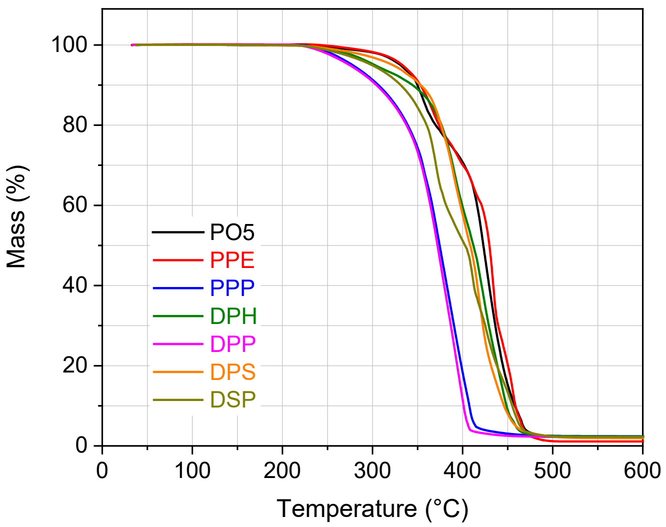

Figure S1. Thermogravimetric analysis of P05 PE-LD and PPE, PPP, DPH, DPP, DPS and DSP mixed PO regranulates. Thermal degradation properties were typical of PP and PE, indicating negligible aging or well-stabilized material. Onset of thermal degradation was determined by the PP phase and further degradation behaviour by the dominant blend component. PP rich regranulates (PPP, DPH, DPP, DPS and DSP) were hence readily distinguishable from those which contained large quantities of PE (P05 and PPE). The inorganic residues of mixed PO regranulates were 0.9 wt.% for PPE, 1.8 wt.% for DPS, 2.1 wt.% for DSP, 2.2 wt.% for DPP, 2.3 wt.% for PPP and 2.4 wt.% for DPH. P05 PE-LD regranulate had an inorganic residue of 2.8 wt.%. Inorganic residues mainly consisted of Si, Ca, Ti, Mg, Fe, S, Na and Cl.

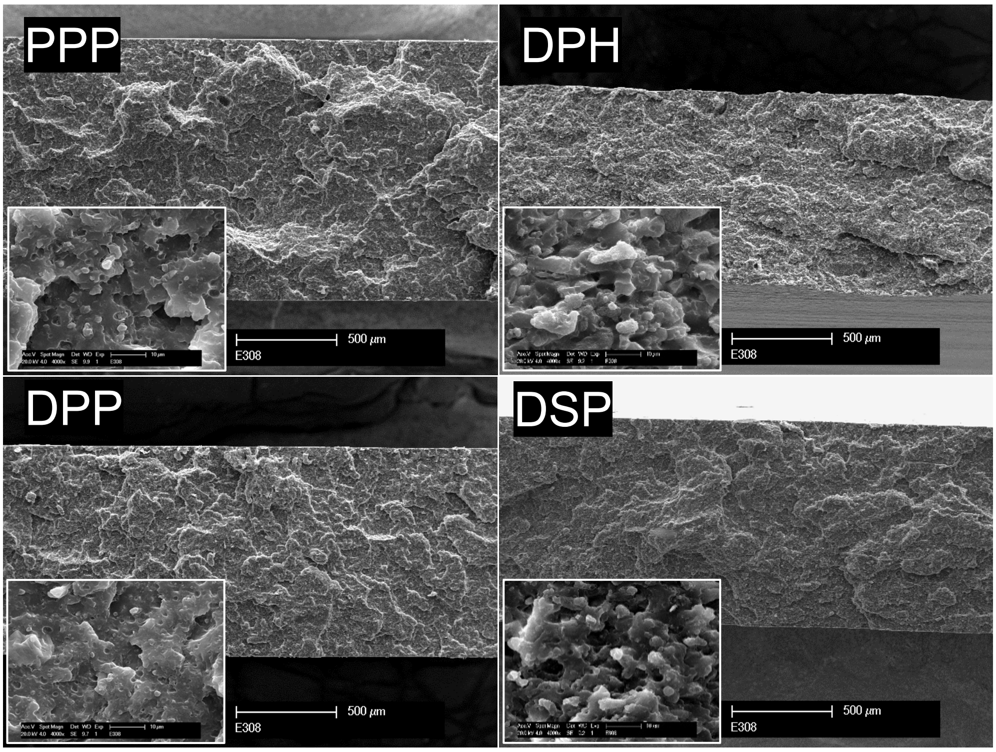

Figure S2. SEM micrographs of the tensile impact fracture surfaces of PPP, DPH, DPP and DSP mixed PO regranulates. Higher magnification micrographs illustrating the phase morphology are provided in the insets.
